# Concurrent Chemoradiation Affects the Clinical Outcome of Small Bowel Complications Following Pelvic Irradiation: Prospective Observational Study from a Regional Cancer Center

**DOI:** 10.7759/cureus.2317

**Published:** 2018-03-13

**Authors:** Velagala Jayapala Reddy, Sathasivam Sureshkumar, Chellappa Vijayakumar, Anandhi Amaranathan, Sundaramurthi Sudharsanan, Prem Shyama, Chinnakali Palanivel

**Affiliations:** 1 Surgery, Jawaharlal Institute of Postgraduate Medical Education and Research (JIPMER), Puducherry, India.; 2 Radiation Oncology, Jawaharlal Institute of Postgraduate Medical Education and Research (JIPMER), Puducherry, India.; 3 Preventive Medicine, Jawaharlal Institute of Postgraduate Medical Education and Research (JIPMER), Puducherry, India.

**Keywords:** radiation enteritis, ileal stricture, radiotoxicity, concurrent chemo radiotherapy, pelvic irradiation, radiotherapy complications

## Abstract

Background

To appraise the spectrum of small bowel complications following pelvic irradiation and to assess the clinical outcome and factors associated with adverse clinical outcome in these patients.

Methods

This descriptive clinical study was done for three years in a tertiary care center in South India. Patients managed for post-irradiation small bowel complications, irrespective of the indication for radiotherapy, were studied. Patients with associated non-gastrointestinal radiation toxicity, radiation proctitis, and radiation colitis were excluded. The parameters assessed were the range of small bowel complications, a comparison of operative and non-operative management, morbidity and mortality, the severity of complications in relation to the dose of radiotherapy, and various factors influencing the clinical outcome.

Results

A total of 50 patients were studied. Stricture and perforation peritonitis were the most common presentation (n=25; 50%). A majority of the patients (n=37; 74%) presented after six months following radiotherapy. Post-operative mortality was 16% (n=5). Age, body mass index (BMI), previous surgery, operative intervention, primary or adjuvant radiotherapy, concurrent chemoradiotherapy (CCRT), and various radiation protocols were not associated with adverse clinical outcomes with respect to overall mortality, the requirement of surgery, and operative mortality. However patients who were operated and those who received CCRT had a significantly longer mean intensive care unit (ICU) stay (3.51 days vs. 0.68 days; p = 0.0001) as well as overall mean hospital stay (14.87 days vs. 5.58 days; p = 0.001) and an insignificant mortality rate (16% vs. 15%; p = 0.4085).

Conclusion

The present study observed that the patients who were operated and those who received CCRT had significantly longer hospitalization and relatively higher mortality. Considering the fact that many of the patients who develop post-irradiation complications may not report back to the same center, the incidence of small bowel complications could be higher in reality, which ascertains the necessity for more precision in the radiation technique and operative care in developing countries.

## Introduction

Pelvic irradiation is one of the principal modes of treatment for both gynecological and non-gynecological malignancies. Radiation oncology has made tremendous progress in terms of newer techniques like high-precision radiotherapy, three-dimensional conformal radiotherapy, intensity modulated radiotherapy, etc. Recent advances in radiation delivery using linear accelerators with novel fractionation schemes and the use of radioprotective drugs during radiotherapy have helped in reducing the frequency and severity of radiotoxicity [1]. In spite of these recent advances, radiation enteritis continues to remain a major clinical problem with an incidence ranging from 0.5% to 5 % [2-3].

The small intestine is the most susceptible organ affected by chronic radiation enteritis and accounts for significant morbidity and a fourfold increase in the mortality compared to radiation colitis or proctitis [4]. The range of small bowel complications and severity may be influenced by many factors, including body mass index (BMI), the dose of radiotherapy, previous abdominal surgery, and associated co-morbidities [2,4]. Surgical treatment for this intestinal radiation injury includes adhesiolysis, bowel resection, and diverting stomas [5].

Considering the fact that about one-third of the patients with chronic radiation enteritis will require surgical treatment ultimately, a detailed analysis of this clinical spectrum is required. Studies on the outcome of small bowel complications following pelvic radiotherapy are also sparse. Hence, this study was carried out to investigate the spectrum and clinical outcome of these complications and factors that significantly influence the outcome in a tertiary care regional cancer center.

## Materials and methods

The study was a descriptive clinical study of patients who were managed for small bowel complications following pelvic radiotherapy. Patients managed for post-irradiation small bowel complications in the department of surgery for three years were assessed for outcome parameters. All patients treated for the small bowel complications following pelvic irradiation irrespective of the indication of the radiotherapy were included in the study. Indications for radiotherapy included gynecological malignancy, prostatic cancer, and carcinoma rectum. Patients with associated non-gastrointestinal radiation toxicity, radiation proctitis, and radiation colitis were excluded from the study.

Written informed consent was obtained from all prospective patients. The investigation and management of the patients presenting to the surgical unit, including the decision to operate or to manage conservatively, were left to the treating surgeon per the existing protocol and depending on the patient condition. All operations were carried out by competent surgeons with reasonable expertise in emergency surgical management. Post-operatively, patients were followed for up to 30 days for outcome parameters, including surgical site infection (SSI). The clinical presentation of post-radiotherapy small bowel complication was categorized into three groups based on the time interval between the radiotherapy and the onset of small bowel complication. The presentation was termed "acute" if a complication develops within six weeks, "late" when presented between six weeks to six months, and "chronic" if more than six months.

The parameters assessed were the range of early and late small bowel complications following pelvic irradiation, the proportion of patients requiring operative management, a comparison of operative and non-operative management, patients requiring more than one surgery, and the reason for the reoperation. This study also analyzed morbidity and mortality, the severity of complications in relation to the dose of radiotherapy, the time interval for the development of a complication after radiotherapy, factors influencing the severity of complications like co-morbidities, BMI, and previous abdominal surgery. The institute's ethics committee approved the study.

Statistical analysis

The distribution of data on the clinical/surgical profile of complications and the outcome were expressed as frequencies and percentages. The comparison of profiles between the subgroups (gender, histological grade, clinical stage, etc.) was carried out using the chi-square test and Fischer’s exact test. To assess the clinical and demographical factors associated with the outcome, a logistic regression analysis was carried out at the 5% level of significance and a P value < 0.05 was considered significant. A statistical analysis was performed using the Statistical Package for Social Sciences (SPSS) 16.0 software (IBM (SPSS) Statistics Armonk, NY).

## Results

In this study, a total of 50 patients with small bowel complications following pelvic radiation for various pelvic malignancies were assessed for the outcome parameters, including the clinical and operative outcome. Out of the 50 patients, 47 patients were female (94%). The age of the patients ranged from 30-70 years with more patients in the age group of 40-60 years. However, the age and gender distribution did not show any significant difference in incidents of post-radiotherapy small bowel complications. In a majority of the patients, the indication of pelvic irradiation was carcinoma cervix representing 90% (n=45) of the study population followed by carcinoma rectum (n=4; 8%) and carcinoma prostate (n=1; 2%). A majority of the study population (90%) had normal BMI. Diabetes and hypertension were present in 28% (n=14) and 4% (n=2), respectively, and 24% (n=12) of patients had both. BMI or presence of co-morbidity did not significantly affect the clinical outcome of small bowel complications.

Small bowel complications: presentation and clinical outcome

Patients admitted for post- pelvic radiation small bowel complications stayed in the hospital for an average of 13.8 days. There was a significant increase in the duration of hospital stay in patients who were operated compared to those who were managed conservatively both in terms of ICU stay (3.51 vs. 0.68; p=0.0001) as well as overall hospital stay (14.87 vs. 5.58; p=0.001) Patients in this study group had a wide range of small bowel complications following pelvic radiotherapy. Mortality in this study group was 16% (Table 1).

**Table 1:** Comparison of baseline parameters in operated and non-operated patients BMI: body mass index; RT: radiotherapy; ICU: intensive care unit; DM: diabetes; HTN: hypertension; h/o: history of; no: number

Parameters	Operated (n=31)	Non operated (n=19)	p-value
Mean age (years)	49.23	50.74	0.633
Mean BMI ( kg/m^2^ )	22.38	22.18	0.796
Mean duration of RT(days)	40.0	39.89	0.839
Mean ICU stay (days)	3.51	0.68	0.0001
Mean hospital stay (days)	14.87	5.58	0.001
DM (no. (%))	9 (29%)	5 (26%)	0.835
HTN (no. (%))	0	2 (11%)	0.065
Both (no. (%))	6 (19%)	6 (32%)	0.329
Past h/o surgery (no. (%))	24 (77%)	15 (79%)	0.899
Mortality (no. (%))	5 (16%)	3 (15%)	0.4085

Stricture and perforation peritonitis, being the most common presentation, occurred in 50% of the patients (n=25), followed by adhesive intestinal obstruction (n=14; 28%), radiation enteritis (n=6; 12%), sub-acute intestinal obstruction (n=4; 8%), and pyoperitoneum (n=1; 2%). The time interval from the radiotherapy to the presentation of surgical small bowel complication ranged from three weeks to 13 years with a mean interval of 32 weeks. A majority of the patients (n=37; 74%) presented after six months. One patient presented early, with perforation peritonitis within six weeks of completion of radiotherapy (n=1; 2%). Twelve patients (24% of the study population) presented late, between six weeks to six months from radiotherapy. Among the patients who received conservative management, eight patients (two with radiation enteritis, two with sub-acute obstruction, and four with acute intestinal obstruction) eventually required surgical intervention.

Out of 31 patients who were operated, all of them had the involvement of the ileum in the form of Ileal perforation (n=21; 67%), stricture, or both (n=12; 39%). One patient (3%) had six perforations in the ileum. The most common operative procedure executed was resection and Ileostomy (n=25; 80.6%). Two patients underwent resection and anastomosis (6.24%). Wedge resection and primary closure were done in one patient (3.2%). Adhesiolysis was done in one patient (3.2%). Laparotomy and lavage were done for pyoperitoneum in two patients (6.24%). Surgical site infection (SSI) (n=11; 35.5%), postoperative pneumonia (n=3; 9.6%), stoma-related complications (n=5; 16%) and deep vein thrombosis (n=1; 3.2%) were the complications seen in the operated patients. In patients with SSI, five (16%) developed laparostoma. One patient (3.2%) developed short bowel syndrome due to extensive resection. None of the patients in the study group had fistulas or hemorrhage as a complication of radiation. Mortality among the patients who underwent various surgical procedures did not differ significantly (16% vs. 15%; p = 0.4085) (Table 2).

**Table 2:** Range of small bowel complications and management in study patients RT: radiotherapy; Perf: perforation; Int: intestinal; SA: sub-acute; no: number

Diagnosis at presentation (n=50) (no. (%))	Time duration between RT and complication			Management	
	Acute (n=1)	Late (n=12)	Chronic (n=37)	Operated (n=31)	Non-operated (n=19)
Perf.Peritonitis (25(50%))	1(100%)	5(41.7%)	19(51.4%)	23(74.4%)	2(10.6%)
Int.Obstruction (14(28%))	0	2(16.7%)	12(32.4%)	3(9.6%)	11(57.9%)
Pyoperitonum (1(02%))	0	0	1(3.2%)	1(3.2%)	0
Radiation enteritis (6(12%))	0	4(33.3%)	2(6.4%)	2(6.4%)	4(21%)
SA Int.Obstruction (4(8%))	0	1(8.3%)	3(9.6%)	2(6.4%)	2(10.5%)

In a subset analysis, though not statistically significant, the patients who underwent surgery following failed conservative management had a relatively longer stay than patients operated immediately after presentation (24.35 days vs. 20.35 days; p = 0.2865).

Factors affecting clinical outcome

A majority of the patients (n=40; 80%) in the study group received primary radiotherapy and 10 patients (20%) received adjuvant radiotherapy following surgery. There were no significant differences in the requirement of operative intervention, overall mortality, and postoperative mortality between the two groups (Table 3).

**Table 3:** Primary vs. adjuvant radiotherapy: comparison of clinical outcome parameters in operated patients RT: radiotherapy

Radiotherapy ( n=50)	Operated (n=31)	Non-operated (n=19)	p-value	Total deaths (n=8)	p-value	Operative mortality	p-value
Primary RT (40(80%))	26(83.9%)	14(73.7%)	0.3821	7(87.5%)	0.6154	5(71.4%)	0.9739
Adjuvant RT (10(20%))	5(16.1%)	5(26.3%)		1(12.5%)		1 (100%)	

Similarly, various clinical presentations, the requirement for operative intervention, and operative mortality did not significantly differ in patients who received various radiotherapy protocols both in carcinoma cervix and other diagnoses (Table 4, Table 5).

**Table 4:** Comparison of radiotherapy protocol and small bowel complications in study patients Ca: carcinoma; RT: radiotherapy; Perf: perforation; SA: sub-acute; Int: intestinal

Diagnosis at presentation (n=50)	Ca.Cervix (n=45)				Ca.Rectum (n=4)	Ca.Prostate (n=1)	p-value
	RT protocol followed (no. (%))						
	1 (n=18)	2 (n=2)	3 (n=7)	4 (n=18)			
Perf.Peritonitis (n=25)	8(32%)	1(4%)	4(16%)	10(40%)	1(4%)	1(4%)	0.7633
Int.Obstruction (n=14)	4(16%)	0	3(12%)	6(24%)	1(4%)	0	0.7660
Pyoperitoneum (n=1)	1(100%)	0	0	0	0	0	0.8742
Radiation enteritis (n=6)	4(67%)	1(16.5%)	0	1(16.5%)	0	0	0.2312
SA Int.Obstruction (n=4)	1 (25%)	0	0	1(25%)	2(50%)	0	0.0565
Mortality (n=8)	1(6%)	0	2(29%)	4(22%)	0	1(100%)	-

**Table 5:** Comparison of radiotherapy protocol and clinical outcome parameters in patients with small bowel complications following pelvic irradiation RT: radiotherapy; no: number; ca: carcinoma

Ca.Cervix (RT protocol)	Operated [no.(%)]	Mean duration of RT (days)	Mean hospital stay (days)	Operative mortality( no. (%))	p-value
Protocol 1 (n=18)	11 (61%)	39.83	9.6	1 (9.1%)	0.4384
Protocol 2 (n=2)	1 (50%)	36	13	0	0.6734
Protocol 3 (n=7)	5 (71%)	39.86	10.71	1 (20%)	0.8812
Protocol 4 (n=18)	12 (67%)	39.94	14.5	3 (25%)	0.4387

On comparing patients who received concurrent chemoradiotherapy (CCRT) and those who received radiotherapy alone, the length of hospitalization was significantly longer (16.94 days vs. 12.38 days; p=0.014) in the concurrent chemoradiotherapy group that was operated (Table 6).

**Table 6:** Comparison of clinical outcome parameters in patients with concurrent chemoradiotherapy (CCRT) with radiotherapy (RT) alone CCRT: concurrent chemo radiotherapy; RT: radiotherapy; no: number

Parameters	CCRT (n=27)		RT (n=23)		p-value
	Operated (n=17)	Non operated (n=10)	Operated (n=14)	Non operated (n=9)	
Mortality (no. (%))	5 (29.4%)	2 (20%)	1 (7.14%)	0	0.5371
Mean hospital stay (days)	16.94	4.7	12.38	6.56	0.014

Factors that can possibly affect the clinical outcome, including age, BMI, previous surgery, operated, conservative management, primary or adjuvant radiotherapy, CCRT, and various radiation protocols, were analyzed for clinical outcomes with respect to overall mortality, requirement for surgery, operative mortality, and length of hospitalization. Patients who were operated and those who received CCRT had significantly longer hospitalization. Other outcome parameters did not differ significantly in these groups.

## Discussion

Radiotherapy is an important treatment modality for both gynecological and non-gynecological pelvic malignancies either as a primary therapy or as an adjuvant therapy after surgery. This study tried to introspect on the range and clinical outcome of small bowel surgical complications following radiotherapy.

Irrespective of improved radiation techniques and safety methods, the incidence of intestinal radiation injury (IRI) remains high. Turina M et al. reported 1.2% to 37% incidence of intestinal complications following pelvic irradiation [1]. In this study, the incidence of small bowel complication was 1.4%. The low incidence could be due to the inclusion of only small bowel complications in the study and improved radiotherapy technique in the regional cancer center.

The most susceptible organ for radiation injury following pelvic irradiation is the small intestine. Rapidly dividing mucosal crypt cells, which are highly radiosensitive, make the small intestine more susceptible to developing post-irradiation complications [6-7]. An acute complication occurs shortly after irradiation and usually resolves in two to six weeks, most of which is dose-dependent. Nearly 50% of the patients develop acute complications if the irradiation dose exceeds 65 Gy. In this study population, the majority (n=37; 74%) presented after six months following radiotherapy and 63% of these patients (n=23) required surgical intervention. This observation was also made by Hatcher et al. and Turina M et al. in their study, where a majority of the complications (80%) was chronic and presented after six months [1,8].

Huscher A et al. described that the incidence of radiation injury to the bowel was more common in patients who received radiotherapy for carcinoma cervix [1,4]. In this study, 45 patients (90% of the study population) who presented with post-radiotherapy small bowel complications had received pelvic radiotherapy for carcinoma cervix. Four patients (8%) had received pelvic radiation for carcinoma rectum and one patient (2%) for carcinoma prostate. Studies from western countries have reported more intestinal complications following radiation for colorectal and prostatic malignancies. Hatcher et al. reported 71 cases with intestinal radiation injuries involving both the small and large intestines. Half of the patients in this study (50%) received radiotherapy for carcinoma cervix [8]. In this study, the clinical presentation ranged from mild radiation enteritis to perforation peritonitis and pyoperitoneum. The most common presentation was perforation peritonitis, which was seen in 25 (50%) of the total study population. Acute intestinal obstruction (n=14; 28%) and sub-acute obstruction (n=4; 8%) were the other common presentations. Hatcher et al. reported that strictures and fistulas were more common due to post radiation intestinal damage and in their study, only six out of 71 patients had intestinal perforations [8]. However, their study included both large and small bowel complications, where a small bowel radiation injury was recorded in only nine patients and six of them had perforations (67%) [8].

Operative intervention

In the study population, 62% of the patients (n=31) had undergone emergency surgical treatment. Surgery in this scenario is challenging because of various factors like post-radiation skin changes, intestinal adhesions, fibrotic changes in mesentery and peritoneum, and intestinal fibrosis and strictures. The most common indication for surgery in this study was the perforation of the small bowel (n=27; 87%). Intestinal obstruction was the most common indication in the study by Regimbeau et al. [2].The reason for this variation could be that, in their study, both small bowel and large bowel complications were included, and it is known that intestinal obstruction is a common presentation of a large bowel radiation injury.

The most common operative finding was an ileal perforation with or without stricture ((n=12; 38.7%) and (n=15; 48.38%)). Stricture alone was seen in one patient (3.2%). The most common operative procedure carried out was the resection of the involved bowel segment and stoma. This was in contrast with other studies by Regimbeau et al. and Hatcher et al., where resection anastomosis was the frequently done surgical procedure [2,8]. In this study, a perforation with a contaminated peritoneal cavity was the common finding, and most of the patients showed evidence of sepsis and required a damage control procedure with a shorter operative time.

Resection and anastomosis were done in two of the operated patients without an anastomotic leak postoperatively (6.4%). Regimbeau et al. reported higher leak rates (10%) after resection and ileoileal anastomosis [2]. Though the number of anastomotic procedures in this study was too low to make any comparison, the observation by Denham JW et al. could possibly explain the higher leak rate, where it was suggested that radical resection may be indicated even with a normal appearing bowel since there can be microscopic pathology, which can progress if left unresected [9]. Hatcher et al. reported that almost 30% of their operated patients required re-surgery [8]. None of these study patients required a second surgery. This may be partly due to the shorter median follow-up. The mortality rate in the operated patients was 16% (n=5). All those operated were emergency patients and all died of sepsis. Regimbeau et al. reported a mortality of 11% in their patients operated in an emergency and all of them died of anastomotic leak and sepsis [2].

Predictors of outcome in post-radiotherapy small bowel complications

Many authors have reported a relation among age, BMI, CCRT, radiation dose, previous abdominal surgery, and co-morbidities with the occurrence and severity of radiation-induced intestinal damage [2,10]. Old age was found to be an important risk factor in determining the severity and outcome of radiation bowel injury [5,11]. In this study, the average age of the patients was 49.2 years. More deaths were observed in patients operated at more than 50 years; however, the difference was not statistically significant.

Iraha S et al. documented that co-morbidities like diabetes mellitus, hypertension, and vascular diseases were associated with more severe radiation enteritis [12]. In this study, even though 56% of the patients (n=28) had co-morbidities at presentation, there was no significant association with the outcome in terms of operative mortality; however, these patients had longer hospital stays than those who did not have any associated co-morbidities. Thin physique was documented as a predisposing factor for radiation enteritis in the study done by Theis et al. [13]. In the present study, BMI did not significantly influence the duration of hospital stay, the requirement of operative management, or mortality. This could be due to the fact that a majority of the study patients were of normal BMI. Complications and mortality were higher in patients with high BMI.

A majority of the patients (n=24; 77%) in this study had undergone surgery prior to the pelvic radiation. However, it did not affect the presentation or operative outcome in these patients compared to those who had no surgical procedures done earlier. Iraha et al., Hatcher et al., and many others, in their studies, showed that the severity of the complication was influenced by prior abdominal surgery [8,12]. Most of the study patients had tubectomy (n=21; 90%), which is a rather minor surgical procedure and is usually carried out without much intra-abdominal manipulation. This could be the reason for the insignificant difference in the outcome among these patients.

Radiation dose, technique, and concurrent chemoradiation

All the patients in this study received a radiation dose of 45 to 50 Gy on an average, as reported by Turina et al [1]. All the patients received almost uniform fractionation schedules with minor variations. There was no significant difference in the outcomes with radiation dose and fractionation schedules in this study. Mersh TG et al. reported that ileal damage was invariably associated with external beam radiotherapy [14]. In this study, all the operated patients were found to have ileal involvement. The administration of chemotherapy along with radiation therapy has shown to correlate with an increased incidence of radiation-related intestinal damage by Jensen H et al. [15]. In this study, seven out of eight patients who expired due to radiation bowel damage had received CCRT, representing 87.5% of the total mortality. However, no statistically significant difference was found in the incidence, clinical outcome, and range of small bowel complications in these patients.

Mortality and secondary cancer

A secondary malignancy in the irradiated area is one of the concerns in patients undergoing pelvic irradiation. Turina et al. reported a 10% incidence of secondary cancer in their patients [1]. None of the patients in this study group had developed secondary cancers in the irradiated area; the reasons could be that the median follow-up period in this study was two years, which was too short to find such an occurrence, and a majority of patients in this study received radiation for carcinoma cervix, which responds to radiotherapy better, with a lower recurrence than rectal and prostatic malignancy, which were the common cases in other studies. The overall mortality in this study was 16% (n=5). A similar range was reported by Turina et al. and many others [1].

## Conclusions

Age, BMI, previous surgery, operated or conservative management, primary or adjuvant radiotherapy, CCRT, and various radiation protocols were not associated with adverse clinical outcomes with respect to overall mortality, the requirement of surgery, and operative mortality in patients who developed post-irradiation small bowel complication. However, patients who were operated and those who received CCRT had significantly longer hospitalization and relatively higher mortality. Considering the fact that many of patients who develop a complication may not report back to the same center, the incidence of small bowel complication could be higher in reality, which ascertains the necessity for more precision in radiation technique and operative care in developing countries like India.
